# Sirolimus-Eluting Stents vs Uncoated Stents for the Treatment of Proximal Left Anterior Descending Coronary Artery Stenosis

**Published:** 2007-12

**Authors:** José Valencia, Vicente Mainar, Pascual Bordes, Alberto Berenguer, Juan Miguel Ruiz-Nodar, Javier Pineda, Silvia Gomez, Francisco Sogorb, Juan Caturla

**Affiliations:** *Servicio de Cardiología, Unidad de Cuidados Intensivos, Hospital General Universitario de Alicante, Spain*

**Keywords:** stent, drug-eluting stents, coronary angioplasty, follow-up studies

## Abstract

Sirolimus-eluting stents (SES) have demonstrated low incidence of target vessel revascularizations in several anatomic scenarios, including proximal left anterior descending coronary artery (pLAD) lesions. The aim of present study was to compare the efficacy of SES with bare metal stents (BMS) for the treatment of such lesions. 96 patients with severe pLAD stenosis treated with SES were included. Clinical follow-up were performed during a 24 month period. A 98 patient sample with pLAD lesions treated with BMS was taken as control group. Death, angiographic restenosis, new target lesion revascularization (TLR) and target vessel failure (TVF) were registered. Clinical, angiographic and procedural variables were analysed to identify predictors of TVF and TLR. Angiographic procedural success was 100% in SES group vs 99% in BMS group (*p*=1.0). At 2.5 years, the cumulative rate of TVF was 9.4% in SES group vs 16.3% in BMS group (*p*=0.15), and the rate of TLR was 5.2% in SES group vs 12.2% in control group (*p*=0.08). The probabilities of cumulative TVF and TLR free survival were in BMS group 83.7% and 87.8%, and in SES group 90.6% and 94.8%, respectively. After multivariate analysis only SES utilization was found as independent protective factor against TVF and TLR (HR 0.38, 95%CI [0.15-0.94] *p*=0.037 and HR 0.21, 95%CI [0.06-0.66] *p*=0.008, respectively), and diabetes as independent predictor of TFV and TLR (HR 2.37, 95%CI [1.07-5.24] *p*=0.034 and HR 3.57, 95%CI [1.29-9.87] *p*=0.014, respectively). This study demonstrates that SES utilization is safe and effective in the tretament of pLAD lesions with a better clinical outcome than BMS in a long-term follow-up.

## INTRODUCTION

Proximal left anterior descending coronary artery (pLAD) lesions represents a special subgroup of ischemic heart disease, given the high-risk profile that these lesions have alone or in the context of multivessel disease (1). The quantity and quality of myocardium at risk, which depends on the pLAD permeability, makes a more aggressive therapeutic approach necessary. Stenting has revealed itself as a first option therapy if feasible for the treatment of these lesions (2, 3, 4, 5, 6). Nevertheless, restenosis has become the Achilles heel for stenting. Neointimal proliferation after successful bare metal stent (BMS) implantation in native coronary arteries can promote the appearance of new lesions, implying the necessity of a new revascularization procedure. The main mechanism of production of such lesions is the smooth muscle cell migration from the media to the intima as a response to the injury that angioplasty with balloon and stenting cause after coronary intervention (7). The incidence of restenosis after conventional stent implantation varies between 19 and 31%.

The main contribution to reduce the restenosis incidence has been the development of drug-eluting stents (DES). These devices release a drug, most commonly antimitotic drugs, through the intima to the media to achieve the objective of reducing the neointimal proliferation. Sirolimus-eluting stents (SES) were the first to demonstrate the efficacy of this kind of stents in humans by means of reducing the restenosis a new revascularizations rate (8). The sirolimus or rapamycin (9) is a natural fermentation compound produced by the fungus *Streptomyces higroscopicus.* It’s a macrolide antibiotic with powerful immunosuppressor activity. It blocks the proliferation and migration of smooth muscle cells by means of stopping the G1-S phase of the mitosis cycle (10). RAVEL study (11) was the first to demonstrate the superiority of SES vs BMS in a randomized clinical trial in favourable selected coronary lesions. Several studies have demonstrated the safety and efficacy of drug eluting stents for the treatment of many anatomic and clinical scenarios, including pLAD stenosis (12, 13). The aim of this study was to compare the long-term results of SES vs BMS for the treatment of pLAD lesions.

## METHODS

### Patient population

This study was a case-control design. Between May 2002 and August 2003, 96 consecutive patients with significant pLAD stenosis were included in the study. These patients came from a larger registry of patients with complex coronary disease treated in our institution between May 2002 and August 2003 with SES and both clinical and angiographic follow-up were performed. Methodology and results of this registry have been reported previously (14). This SES-group was compared to 98 consecutive patients with pLAD lesions treated with BMS in our laboratory between April 1995 and April 1998 (at that period DES were not available). Characteristics of this population and its outcomes have been widely described previously (15).

### Inclusion and exclusion criteria

Patients with significant pLAD stenosis (stenosis > 70% by visual estimation) and demonstrable ischemia of LAD dependent myocardium were included. Patients with acute myocardial infarction (primary angioplasty), cardiogenic shock, and life expectancy lower than one year were excluded. All patients initially assessed for inclusion into the study underwent coronary angioplasty and subsequent planned clinical follow-up.

### Coronary stent procedure

All patients signed an agreement form just before procedure. All procedures in the SES group were performed according to the standard interventional techniques (16) and to the recommendations concerning the specific use of drug-eluting stents (17). SES used was Cypher^®^ (Cordis, Johnson & Johnson, USA). Periprocedural medications including glycoprotein IIb/IIIa blockers, direct stenting technique or postdilatation with a balloon 0.5 mm larger than the stent diameter, were left to the operator’s discretion. “Angiographic success” was defined as residual stenosis <30% with TIMI 3 flow. All patients in SES group received a loading dose of 300 mg clopidogrel and then 75 mg/d for 6 months in addition to 100-250 mg/d aspirin.

### Follow-up and clinical events

Clinical follow-up was evaluated 1, 6, 12 and 24 months after procedure by office visits or telephone interviews. The incidence of death, target vessel myocardial infarction (TVMI), defined as an elevation of creatine kinase at least twice as high as the normal upper limit with ECG changes involving anterior leads, new target lesion revascularization (TLR) and target vessel failure (TVF, composed end-point including death, LAD dependent MI or TLR) were registered. Definitive stent thrombosis was defined as the presence of acute coronary syndrome with angiographic evidence of stent thrombosis or occlusion in target vessel. Patients in SES group underwent to routine control angiography at sixth month. Control coronariography in BMS group was performed only under clinical indication. Binary restenosis was defined as a >50% diameter stenosis of the target lesion.

### Statistical analysis

Analysis were performed with the SPSS version 11.0^®^ statistical package (SPSS Inc. Chicago, Illinois, USA). Data are expressed as mean ± SD for continuous variables and as frequencies for categorical variables. Differences between SES-group and BMS group were assessed by Student’s *t* test. Discrete variables are reported as frequencies (percentages) and compared by Chi-square’s or Fisher’s tests (were appropriate). All tests were two-tailed and a *p* value <0.05 was considered as statistically significant. Multivariate Cox regression analysis was performed to identify independent predictors of TVF and TLR. All variables analysed in the univariate analysis were included in the multivariate analysis. A backward conditional stepwise method was used to perform the variable inclusion in the model. Probability values for stepwise were 0.5 for entry and 0.10 for removal with a maximum of iterations of 20. Hazard ratios (HR) with CI at 95% were estimated. Assessment of the linearity assumption for continuous variables was tested with a *martingale* residues analysis. Survival-free curves of TLR and TVF were calculated according to the Kaplan-Meier method and log-rang test was used to compare TVF and TLR incidence between SES and BMS groups.

## RESULTS

### Baseline and procedural characteristics

Baseline clinical and procedural characteristics of SES and BMS groups are shown in Table 1. Compared to BMS group, SES group had more multivessel disease, more active smokers and less hypercholesterolemia. SES group also had more ostial lesions, required more stents with longer length and fewer diameter of them. There was no difference in diabetic proportion. A more extensive analysis of angiographic and clinical results in the SES group has been previously described (18).

**Table 1:** Baseline demographic and procedural characteristics of all patients

	BMS group (n=98)	SES group (n=96)	*p*

**Age (mean ± SD yrs)**	62.5 ± 9.2	62.7 ± 12	0.900
**Female %**	17.3	20.8	0.540
**Hypertension %**	59.2	50.0	0.200
**Diabetes %**	26.5	31.3	0.470
**Hypercholesterolemia %**	81.6	58.3	<0.001
**Current smokers %**	18.4	31.3	0.038
**Multivessel disease %**	28.6	51.0	0.001
**Depressed LVEF %**	30.6	26.3	0.510
**Procedural success %**	99.0	100	1
**Ostial lesion %**	6.1	28.1	<0.001
**Number of stents**	1.1 ± 0.3	1.2 ± 0.4	0.036
**Stent length (mm)**	17 ± 5.2	23.4 ± 11.7	<0.001
**Stent diameter (mm)**	3.4 ± 0.3	3 ± 0.2	<0.001

Depressed LVEF, left ventricle ejection fraction <50%.

### Clinical outcomes

Complete follow-up was available in 99% of all patients (mean follow-up period, 31.5 ± 7.2 months). Incidences of adverse cardiac events in both groups are shown in Table 2. There were no differences between BMS group and SES group either incidence of death or TVMI. There was a strong trend towards worse prognosis in BMS group in terms of TVF and TLR, with a difference nearly statistically significant in TLR (12.2% in BMS group vs 5.2% in SES group, *p*=0.083). SES group also showed less binary restenosis incidence than control group (7.3 vs 19.4, *p*=0.013), although routine control coronariography was performed in 86.5% of SES patients and only in 25.5% of BMS patients (in this group angiography was performed only based on clinical criteria). There were only two cases of definitive stent thrombosis in the SES group. One case nine days after procedure and the other one at fifth month due to premature clopidogrel therapy suspension. No stent thrombosis was detected in BMS group although 2 patients suffered TVMI without control coronariography due to negative myocardial ischemia or viability tests after event.

**Table 2:** Long-term clinical outcomes

	BMS group (n=98)	SES group (n=96)	*p*

**Death %**	5.1	2.1	0.45
**Cardiac death %**	2.0	1	1
**TVMI %**	2.0	5.2	0.28
**TVF %**	16.3	9.4	0.15
**TLR %**	12.2	5.2	0.083
**Angiographic restenosis %**	19.4	7.3	0.013
**Stent thrombosis %**	0	2.1	0.24

**Figure 1:**
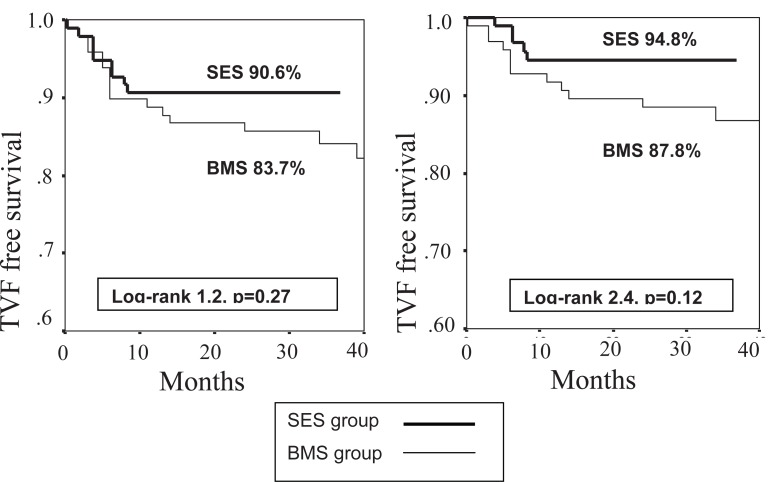
Kaplan-Meier curves for 40 month TVF (left) and TLR (right) free survival in patients treated with sirolimus-eluting stents (SES group) and bare metal stents (BMS group).

At 40 month, the cumulative TVF and TLR free survival were 83.7% and 87.8% for BMS group, and 90.6% and 94.8% for SES group (Figure 1). Log-rank tests did not show statistically significant differences between both groups although SES patients had overall better outcomes in these end points.

### Predictors of adverse events

The impact of the different clinical, angiographic and procedural characteristics analysed on the risk of subsequent incidence of TVF and TLR is described in Table 3. Only SES utilization was found as independent protective factor against TVF and TLR (HR 0.38, 95%CI [0.15-0.94] *p*=0.037 and HR 0.21, 95%CI [0.06-0.66] *p*=0.008, respectively), and diabetes as independent predictor of TFV and TLR (HR 2.37, 95%CI [1.07-5.24] *p*=0.034 and HR 3.57, 95%CI [1.29-9.87] *p*=0.014, respectively). Bigger stent diameters presented a strong trend to have better outcomes (OR 0.30, 95%IC [0.08-1.10] *p*=0.070, and OR 0.24 95%IC [0.05-1.17] *p*=0.078, for TVF and TLR respectively).

**Table 3:** Multivariate predictors of TVF and TLR (Cox proportional hazard model)

	TVF			TLR		
	HR	95% CI	*p*	HR	95% CI	*p*

**SES utilization**	0.38	0.15-0.94	0.037	0.21	0.06-0.66	0.008
**Diabetes**	2.37	1.07-5.24	0.034	3.57	1.29-9.87	0.014
**Stent diameter > 3 mm**	0.30	0.08-1.10	0.070	0.24	0.05-1.17	0.078
**Stent length**	1.02	0.98-1.07	0.28	0.99	0.93-1.07	0.99
**Age**	0.99	0.95-1.04	0.68	0.99	0.94-1.06	0.94
**Ostial lesion**	1.43	0.43-4.76	0.56	1.10	0.20-5.93	0.91
**Female**	1.11	0.39-3.14	0.84	1.12	0.33-3.76	0.86
**Hypertension**	1.62	0.69-3.79	0.26	2.33	0.75-7.22	0.14
**Hypercholesterolemia**	0.91	0.36-2.34	0.85	0.85	0.27-2.68	0.78
**Current smokers**	0.78	0.46-1.33	0.37	0.55	0.27-1.12	0.10
**Multivessel disease**	1.12	0.45-2.75	0.81	1.41	0.49-4.06	0.52
**Depressed LVEF**	0.60	0.23-1.56	0.29	0.22	0.09-1.31	0.12

Depressed LVEF, left ventricle ejection fraction <50%.

## DISCUSSION

The present study found that SES utilization for the treatment of patients with pLAD stenosis was safe, as seen before with BMS stents, and had fewer incidences of adverse cardiovascular events as TVF and TLR than patients treated with uncoated stents. Lack of statistically significant differences found in the incidence of TVF and TLR after univariate analysis can be explained by the insufficient sample size and, fundamentally, the worse lesion profile in the SES group, with more ostial lesions, smaller vessel diameter with smaller stents required and the longer stent utilization in SES group than in control one. A better matched control group would necessitate reducing the sample size and, therefore, the statistical power of the multivariate analysis. Nevertheless, the multivariate analysis confirmed that SES implantation was the unique factor associated with better outcomes in terms of less TVF and TLR incidence. Diabetes, a well known risk factor for adverse cardiac events after stenting (19), even with SES (20, 21), was confirmed as a negative prognostic key.

There are few reports regarding treatment of pLAD stenosis either with drug eluting stents or with a follow-up longer than one year. In a substudy of the SIRIUS trial by Sawhney *et al* (7), 68 patients with pLAD disease treated with SES showed a TVF and TLR incidence of 10.4% and 9%, respectively. TVF value is very similar to 9.4% shown in our study, but TLR incidence found by our group, 5.2%, reduced by 58% the value pointed out by Sawhney *et al*.

In another substudy of the TAXUS-IV trial (8), that included 126 patients with pLAD stenosis treated with paclitaxel-eluting stents, the incidence of TVF and TLR were 13.3% and 6.3%, respectively. These values are also very similar to our results. Seung *et al* (22) demonstrated the effectiveness of SES in 68 patients with the more complex scenario of ostial LAD, compared with a control group treated with BMS. SES implantation was strongly encouraged to be guided with intravascular ultrasound examination for accurate lesion assessment and optimal stenting technique. After one-year follow-up no one of the SES patients required new TLR as compared with the 17% of TLR in the control group. Only a 5.1% of binary restenosis was found in the SES group vs 32.3% in the control group (*p*<0.001).

The results obtained with these devices may be comparable to those of coronary bypass surgery (23). Although a recent study (24) has reported significant fewer reinterventions in patients treated with off-pump bypass surgery of the LAD compared to patients treated with SES, only one fourth of these revascularizations were in LAD, because of that, authors of this study suggest that reinterventions in SES group may be related to incomplete revascularization and not directly to SES failure.

Binary restenosis was significantly lower in patients treated with SES than in the control group. This is concordant with prior randomized studies (7, 8) comparing uncoated stents vs drug-eluting stents which have shown important benefits in the angiographic parameters of pharmacoactive stents in the follow-up coronariography. Nevertheless, our results have to be taken cautiously because of the lack of routine angiography performed in the control group, as compared to the SES group. On the other hand, control angiography in BMS group was clinically driven. Thus, the true rate of binary restenosis in this group should be higher than described, because of the silent restenosis phenomenon that we were not be able to discover in the control patients, and that can affect approximately half of patients with angiographic restenosis (25).

Stent thrombosis has become the main threat after drug-eluting stent implantation. Published isolated cases of patients suffering MI due to late or very late stent thrombosis, in many patients after clopidogrel discontinuation, have raised the concerns about this item. Late rendotelization induced by the drugs released by the DES can promote stent thrombosis even one year after the procedure. The incidence of stent thrombosis after BMS has been reported to be 1-2% although the majority of theses events occurs during the first month after stenting. Clinical trials with drug-eluting stents have reported very similar rates of incidence but with a great number of late or very late episodes. A recent pooled analysis or randomized trials with SES has detected no differences between control patients and patients treated with SES. The incidence of stent thrombosis was 1.5% in SES group vs 1.7% in controls (26, 27). Our finding of 2.1% of stent thrombosis in pLAD lesions after SES is slightly higher although very similar to previously reported data.

In conclusion, this study shows that pLAD interventions with SES are safe and have better outcomes compared with uncoated stents in a long-term follow-up. In this population, SES was found as the unique predictor of a risk reduction of TVF and TLR.
